# Supplementary material for: Real world federated learning with a knowledge distilled transformer for cardiac CT imaging
Source: NPJ Digit Med. 2025 Feb 6;8:88. doi: 10.1038/s41746-025-01434-3 (PMC11802793; doi:10.1038/s41746-025-01434-3)
Supplement: Supplementary file 1 — Supplemental Material [file 41746_2025_1434_MOESM1_ESM.pdf]

Supplementary Information

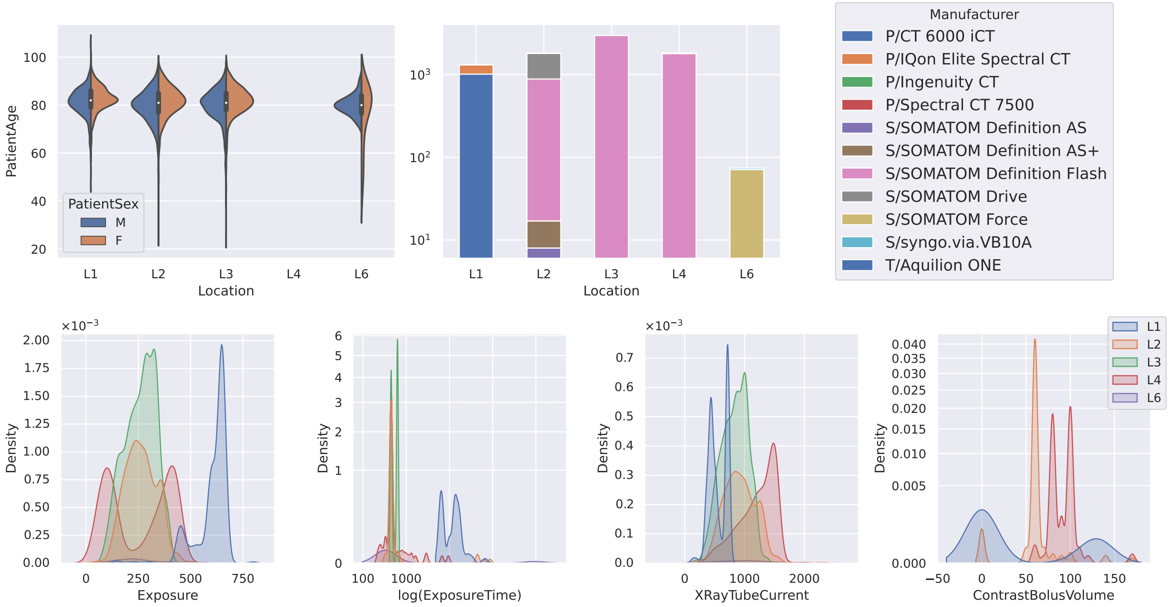

Supplementary Figure 1: **Title:** Demographics of patients and data properties across locations. **Legend:** Some data was not available at all locations. Three manufacturers with in total eleven different models were included in the federated training. The acquisition protocols in terms of exposure, exposure time, X-ray tube current, and contrast bolus volume vary across locations. Manufacture acronyms are P: Philips, S: Siemens, T: Toshiba.

Supplementary Table 1: **Title:** Results of local, federated, and knowledge distilled models per location for the task of detecting hinge points and coronary ostia (HPs & COs).
Legend: Fed and KD are trained on L1,2,3,4,6. The local models often overfit to the training data and even underperform on their respective testset. The federated and especially knowledge distilled models show better generalization. All values are reported in mm with mean and standard deviation.

	Train	L1	L2	L3	L4	L5	L6	L7
UNet	L1	3.58 ± 5.17	3.74 ± 2.03	3.67 ± 1.68	4.35 ± 3.94	3.23 ± 1.83	2.85 ± 1.62	4.84 ± 3.46
	L2	17.15 ± 21.42	11.65 ± 12.59	11.24 ± 11.08	12.31 ± 9.99	12.63 ± 12.29	11.71 ± 11.43	13.56 ± 11.62
	L3	5.98 ± 7.54	4.79 ± 3.2	5.19 ± 8.77	5.01 ± 3.52	4.88 ± 2.17	3.77 ± 2.03	5.35 ± 3.72
	L4	4.81 ± 5.35	4.9 ± 3.19	8.0 ± 10.94	4.53 ± 4.05	4.14 ± 2.3	3.81 ± 5.95	5.44 ± 3.84
	L6	4.58 ± 3.75	4.83 ± 3.74	4.97 ± 3.22	4.53 ± 3.50	3.29 ± 4.32	3.12 ± 3.96	4.73 ± 3.88
	Fed	3.92 ± 5.6	3.53 ± 2.36	3.58 ± 1.79	4.12 ± 4.0	3.41 ± 4.18	2.71 ± 1.28	3.86 ± 3.09
	KD	3.84 ± 5.3	3.77 ± 1.93	3.26 ± 1.78	4.43 ± 4.03	3.37 ± 1.83	2.61 ± 1.24	4.59 ± 3.77
ViT	L1	4.93 ± 5.23	4.7 ± 2.48	3.82 ± 2.11	6.1 ± 3.71	5.1 ± 9.43	3.76 ± 2.16	5.75 ± 3.61
	L2	18.92 ± 21.33	15.45 ± 17.23	14.63 ± 13.21	14.99 ± 12.15	13.72 ± 12.30	15.78 ± 17.48	13.78 ± 14.63
	L3	15.62 ± 26.09	8.65 ± 14.66	7.6 ± 14.63	13.58 ± 21.33	12.68 ± 20.17	9.49 ± 14.03	16.03 ± 24.19
	L4	26.87 ± 7.05	25.24 ± 6.42	24.65 ± 5.02	24.75 ± 10.91	26.06 ± 6.18	23.39 ± 6.5	26.33 ± 5.73
	L6	5.37 ± 34.83	5.11 ± 3.98	5.12 ± 4.87	5.62 ± 3.28	4.05 ± 5.45	6.36 ± 4.05	5.82 ± 3.96
	Fed	4.68 ± 5.26	4.47 ± 2.62	3.75 ± 2.18	5.4 ± 3.81	3.87 ± 1.97	3.09 ± 1.28	5.11 ± 3.53
	KD	4.68 ± 5.25	4.4 ± 2.57	3.69 ± 2.14	5.37 ± 3.81	3.9 ± 1.94	3.07 ± 1.42	5.01 ± 3.43
SWIN	L1	3.61 ± 6.17	3.62 ± 2.22	3.76 ± 1.72	5.8 ± 7.03	2.76 ± 1.47	2.79 ± 1.7	4.29 ± 3.28
	L2	15.93 ± 28.61	3.9 ± 2.01	7.02 ± 8.66	17.9 ± 25.69	15.55 ± 20.56	17.13 ± 12.91	11.47 ± 19.15
	L3	15.86 ± 13.07	9.99 ± 10.17	3.65 ± 5.66	14.09 ± 9.64	8.2 ± 9.96	9.21 ± 11.0	10.49 ± 11.28
	L4	4.66 ± 7.14	3.54 ± 2.27	3.48 ± 1.81	4.84 ± 6.42	3.58 ± 1.83	1.97 ± 1.02	4.17 ± 3.25
	L6	5.12 ± 6.23	3.58 ± 4.83	3.21 ± 2.91	4.58 ± 3.27	2.86 ± 2.75	3.42 ± 2.53	3.92 ± 3.33
	Fed	4.73 ± 7.16	3.47 ± 1.96	3.93 ± 1.85	5.65 ± 6.63	3.02 ± 1.68	2.85 ± 1.49	4.34 ± 3.17
	KD	3.49 ± 5.36	3.26 ± 1.93	3.18 ± 1.78	4.17 ± 3.94	2.94 ± 1.83	2.39 ± 1.11	4.11 ± 3.35

Supplementary Table 2: **Title:** Results of local, federated, and knowledge distilled models per location for the task of detecting the membranous septum (MS).
Legend: Fed and KD are trained on L1 and L3. The local models sometimes overfit to the training data and even underperform on their respective testset. The federated and especially knowledge distilled models show better generalization. All values are reported in mm with mean and standard deviation.

	Train	L1	L3	L7
UNet	L1	3.45 ± 2.63	5.10 ± 0.60	5.01 ± 2.33
	L3	4.68 ± 2.73	3.66 ± 1.06	4.36 ± 1.88
	Fed	4.64 ± 2.33	3.72 ± 1.34	4.37 ± 2.41
	KD	3.26 ± 2.34	3.25 ± 1.32	3.40 ± 1.56
ViT	L1	3.55 ± 2.55	3.29 ± 1.53	4.26 ± 2.65
	L3	54.28 ± 36.64	24.52 ± 18.99	53.98 ± 34.95
	Fed	3.69 ± 2.54	4.49 ± 1.91	5.39 ± 2.64
	KD	3.34 ± 2.39	2.97 ± 1.50	3.60 ± 1.56
SWIN	L1	4.44 ± 3.55	4.75 ± 1.98	4.92 ± 1.63
	L3	3.94 ± 2.33	3.04 ± 0.91	4.60 ± 2.31
	Fed	3.17 ± 2.43	3.30 ± 1.60	3.43 ± 1.44
	KD	3.29 ± 2.44	2.72 ± 0.96	3.29 ± 1.45

Supplementary Table 3: **Title:** Results of local, federated, and knowledge distilled models per location for the task of segmenting the calcification.
Legend: Fed and KD are trained on L1 and L2. The DICE scores are reported with mean and standard deviation.

	Train	L1	L2	L6
UNet	L1	0.593 ± 0.233	0.539 ± 0.134	0.583 ± 0.412
	L2	0.391 ± 0.170	0.401 ± 0.207	0.272 ± 0.274
	Fed	0.486 ± 0.193	0.515 ± 0.246	0.391 ± 0.212
	KD	0.537 ± 0.177	0.500 ± 0.275	0.526 ± 0.228
ViT	L1	0.694 ± 0.136	0.616 ± 0.268	0.663 ± 0.241
	L2	0.378 ± 0.129	0.516 ± 0.209	0.327 ± 0.272
	Fed	0.680 ± 0.138	0.648 ± 0.272	0.636 ± 0.274
	KD	0.569 ± 0.169	0.542 ± 0.248	0.566 ± 0.231
SWIN	L1	0.704 ± 0.138	0.647 ± 0.285	0.661 ± 0.243
	L2	0.384 ± 0.199	0.519 ± 0.236	0.312 ± 0.222
	Fed	0.667 ± 0.155	0.652 ± 0.277	0.682 ± 0.230
	KD	0.652 ± 0.176	0.627 ± 0.273	0.670 ± 0.231
